# Adult Attachment and Personality as Predictors of Jealousy in Romantic Relationships

**DOI:** 10.3389/fpsyg.2022.861481

**Published:** 2022-04-12

**Authors:** Marina Richter, Katja Schlegel, Philipp Thomas, Stefan Johannes Troche

**Affiliations:** ^1^Department of Psychology and Psychotherapy, Witten/Herdecke University, Witten, Germany; ^2^Department of Psychology, University of Bern, Bern, Switzerland

**Keywords:** jealousy, personality, attachment, infidelity experience, gender differences

## Abstract

Functional relationships between romantic jealousy and traits, such as neuroticism or adult attachment styles, are well-known. For the first time, we conducted a joint analysis of the Big Five traits and attachment dimensions as predictors of jealousy, which considered gender differences as well as differences in infidelity experiences and relationship status. In 847 participants, path modeling showed that higher neuroticism, lower agreeableness, and lower openness predicted higher romantic jealousy. The attachment dimensions “anxiety” and “depend” partly mediated the effect of neuroticism and fully mediated the effect of agreeableness on romantic jealousy. The direct and indirect relationships did not differ as a function of gender, relationship status, and infidelity experiences. These findings contribute to a better understanding of individual differences in romantic jealousy from a personality perspective.

## Introduction

Jealousy is a multifaceted emotional, cognitive, and behavioral phenomenon, possibly as old as humanity. It can be observed throughout the lifespan, for instance between siblings for a parent’s attention, in friendships, or in romantic relationships (Hart and Legerstee, 2010). In adulthood, romantic jealousy appears to be the most frequently studied type of jealousy (Harris and Darby, 2010). White (1981) defined romantic jealousy as a “complex of thoughts, feelings, and actions which follows threats to the existence or the quality of the relationship, when those threats are generated by the perception of a real or potential attraction between one’s partner and a (perhaps imaginary) rival” (p. 130). Hence, jealousy is a usually unpleasant but often adaptive phenomenon, with the underlying desire to maintain the relationship with the partner (Harris, 2003).

There are pronounced individual differences in the perception and reaction to this form of rivalry. Psychological research has focused not only on the outcomes of such individual differences for the relationship (e.g., relationship satisfaction or stability; Sheets et al., 1997; Dugosh, 2000), but also on mechanisms and personality correlates underlying those individual differences in romantic jealousy (Buunk, 1982, 1995; Sharpsteen and Kirkpatrick, 1997).

### Attachment and Jealousy

Attachment seems to be the most basic relationship-related personality trait underlying human emotion, cognition, and behavior toward other people. According to attachment theory (Bowlby, 1969, 1982), within the first 18 months of life, infants generate a specific representation of relationships based on their experience of received comfort and relief by their primary caregivers. Depending on the caregiver’s availability, sensitiveness, and responsiveness to the infant’s needs, children develop a secure or an insecure attachment style with a further distinction between avoidant and anxious ambivalent for the latter one (Mikulincer and Shaver, 2007).

Hazan and Shaver (1987) extended attachment theory toward adults to explain individual differences in adults’ experiences, beliefs, and behaviors in close (primarily romantic) relationships. Consistent with the literature on children, they proposed three categories (*secure*, *anxious*, and *avoidant*) and developed a brief vignette measure in which participants choose which of the three categories describes them best. More recently, other researchers have conceptualized adult attachment with continuous dimensions instead of categories and have developed longer questionnaire measures that allow for a more fine-grained measurement of individual differences (for a review see Ravitz et al., 2010). For example, Collins and Read (1990) differentiated between three dimensions—*close* (feelings about comfort with closeness to others), *depend* (dependability on others), and *anxiety* (fear of being left alone or abandoned)—where low values on the first two loosely correspond to an avoidant style and high values on the last one reflect an anxious style.

Harris and Darby (2010) proposed a two-stage model linking adult attachment and jealousy, in which they postulate that securely and insecurely attached individuals differ in how they appraise threat (stage 1) and how they react to threat once another person has been determined as a rival (stage 2). According to their model, securely attached people have a higher threshold to perceive someone else as a rival (i.e., as threatening) than insecurely (anxiously or avoidantly) attached people because they trust their partners more and have lower expectations to be betrayed or abandoned. They thus anticipate feeling less jealous when presented with hypothetical threat scenarios, consistent with the empirical results of Buunk (1997) and Powers (2000), and they also seem to engage less in partner surveillance behaviors (Karakurt, 2001; Marshall et al., 2013). However, once these individuals’ threshold to perceive a rival as threatening has been exceeded, Harris and Darby (2010) predict strong jealous reactions directed at the partner to discourage him or her from engaging with interlopers and to maintain their romantic relationship. Accordingly, Sharpsteen and Kirkpatrick (1997) found that, when recalling actual experiences of jealousy, securely attached individuals’ jealous feelings were as intense as those of insecurely attached individuals but also brought the couple closer together more frequently. In contrast, anxiously attached individuals tend to distance themselves and suppress their anger toward the partner, which might prevent further feelings of rejection (Sharpsteen and Kirkpatrick, 1997; Guerrero, 1998). Karakurt (2001), however, found that anxiously attached people showed more behavioral jealousy than securely attached people. Avoidantly attached people seem to show yet other reactions to threat caused by a rival. They tend to act aggressively toward the rival (as opposed to their partner) and try to induce jealousy in their partner, despite feeling less overall anger than the other two attachment styles (e.g., Powers, 2000; Wegner et al., 2018).

To summarize, past literature suggests that securely attached people are not very jealous before they perceive their relationship to be threatened, but very jealous once they have determined such a threat. Anxiously attached individuals, in contrast, seem to show roughly the opposite pattern and avoidant individuals seem to have less jealous feelings despite reacting to threats with jealousy induction and revenge.

### The Attachment—Jealousy Relationship and the Big Five Personality Traits

Attachment and jealousy can also be examined within the context of the broad personality dimensions neuroticism, extraversion, openness, agreeableness, and conscientiousness, labeled the Big Five (Costa and McCrae, 1992; McCrae and Costa, 2008).

The Big Five play an important role in many areas of life, including the interpersonal domain. For instance, the Big Five predict romantic relationship satisfaction (Malouff et al., 2010), responsiveness, and positive affect in parenting (Koenig et al., 2010), and preferences in friendship formation (Altmann and Roth, 2020). Specifically, low neuroticism emerged as the strongest predictor of high relationship satisfaction and positive parenting behaviors (Koenig et al., 2010; Malouff et al., 2010). In addition, high agreeableness and conscientiousness contributed to higher relationship satisfaction, whereas high extraversion and openness favored positive affect in parenting depending on the child’s temperament (Koenig et al., 2010). In terms of friendship formation, higher openness and conscientiousness predicted a preference for cross-sex over same-sex friendships (Altmann and Roth, 2020).

Considering these associations, surprisingly few studies have examined whether the Big Five predict jealousy. Results showed that neuroticism was relatively consistently related to higher jealousy, with medium effect sizes (Melamed, 1991; Buunk, 1997; Dijkstra and Barelds, 2008; Gehl, 2010; but see Wade and Walsh, 2008). People high in neuroticism may feel more inadequate as a partner and thus feel more easily threatened by potential rivals (Wade and Walsh, 2008; Karakurt, 2012). The other Big Five traits do not seem to substantially affect jealousy, although only very few studies have assessed all five traits (Dijkstra and Barelds, 2008; Wade and Walsh, 2008; Gehl, 2010).

Several studies have also assessed the relationship between the Big Five and adult attachment. Again, the most important predictor of anxious and avoidant attachment was neuroticism, with medium effects (for a review see Noftle and Shaver, 2006), which can be explained by insecurity as the common denominator between these constructs. Overall, openness was unrelated to attachment whereas extraversion, agreeableness, and conscientiousness showed small negative associations with anxious and avoidant attachment (Noftle and Shaver, 2006). These negative associations can be explained by higher assertiveness and confidence of extraverts, higher levels of trust and altruism of agreeable people, and higher levels of self-discipline of conscientious individuals, all of which characterize attachment styles low in anxiety and/or avoidance (Noftle and Shaver, 2006). Conceptually, these same facets may also relate to feeling and/or showing less jealousy.

Taken together, previous research has shown that the Big Five (in particular, neuroticism) predict both adult attachment and jealousy (Buunk, 1997; Noftle and Shaver, 2006), and that attachment predicts jealousy (e.g., Sharpsteen and Kirkpatrick, 1997). However, to date, it remains unclear which set of variables—the Big Five traits or adult attachment—better explains individual differences in jealousy, and whether each set of variables independently contributes to jealousy. On the one hand, the Big Five might be more predictive because they encompass more personality facets relevant to attachment as well as the affective and behavioral elements of jealousy, such as insecurity, trust, confidence, or self-discipline. Given that Costa and McCrae (2012) conceptualized attachment as a set of personality traits that is embedded in and strongly affected by basic personality dispositions, attachment might fully mediate the link between the Big Five and jealousy. On the other hand, adult attachment might be more predictive than the Big Five because like jealousy, both adult attachment and jealousy are focused on romantic relationships. Examining neuroticism, attachment styles, and jealousy, Buunk (1997) reported that attachment was a better predictor of jealousy than neuroticism, and Shaver and Brennan (1992) as well as Noftle and Shaver (2006) found that adult attachment better predicted relationship quality than the Big Five. However, to our knowledge, no study to date has compared and jointly assessed all Big Five traits and attachment as predictors of jealousy. Therefore, the first aim of the present study is to extend the existing research and investigate whether attachment style as a relationship-specific aspect of personality and the Big Five as more general aspects of personality explain unique and/or common portions of individual differences in romantic jealousy.

### Potential Moderators of the Attachment—Jealousy Relationship

The second aim of this study is to test whether the association between attachment, jealousy, and the Big Five is moderated by gender, relationship status, and/or infidelity experience. Many studies have examined gender differences in jealousy, with mixed findings (for a review see Edlund and Sagarin, 2017). To our knowledge, only Buunk (1997) tested whether the association between attachment and jealousy differed by gender and he found no difference. However, a study by Ein-Dor et al. (2015) found that women and men differed in their mate retention strategies, with women being more alert to infidelity cues and more focused on potential rivals and men focusing more on monitoring their partner’s intentions. Applying these results to the first stage of the attachment–jealousy model by Harris and Darby (2010), that is, the threshold for seeing someone as a rival, it might be that securely attached females show higher jealousy than securely attached men because they focus more on potential rivals.

Little is also known about whether relationship status might affect the link between attachment style, Big Five personality traits, and jealousy. Previous studies have shown that people in monogamous relationships report higher feelings of jealousy than singles (e.g., Valentova et al., 2020), whereas adult attachment is considered a more stable personality characteristic that is less likely to change due to relationship status. Self-reported jealousy differences between people in monogamous relationships and singles can emerge when the former group completes a jealousy questionnaire about their current relationship, whereas singles are instructed to refer to a past or imagined relationship. Retrospective and hypothetical jealousy measures tend to yield different results (typically, lower jealousy ratings) compared to those targeting actual or current feelings or experiences (see Edlund and Sagarin, 2017, for a discussion). The association between attachment style and jealousy might be stronger for people in a relationship than for singles, because at the time of the assessment, people in a relationship experience more jealousy-relevant cues and are motivated to maintain their relationship. Thus, the effects of attachment on jealousy might be more prominent than when people are single.

As outlined above, having experienced jealousy-provoking situations in the past (such as partner’s infidelity) might moderate the link between attachment style and jealousy. For example, securely attached individuals with infidelity experiences might lower their threshold for perceiving someone else as a rival and become more jealous compared to those without such experience (in order to lower the risk of future partner infidelity). On the contrary, anxious people might become more detached and less jealous when realizing that surveillance and high jealousy did not actually prevent infidelity. To our knowledge, this question has not been examined to date, although several studies have investigated the direct association between actual infidelity experience and jealousy without considering attachment style (e.g., Tagler, 2010; Varga et al., 2011).

### The Present Study

In order to investigate the two aims of the present study—comparing the Big Five personality traits and attachment as predictors of jealousy; and assessing gender, relationship status, and infidelity experience as moderators of the relationships between jealousy, personality traits, and attachment dimensions—we administered measures of all three constructs to a large community sample of participants in Germany and Switzerland. Many previous studies on the link between romantic jealousy and attachment (Buunk, 1997; Sharpsteen and Kirkpatrick, 1997; Guerrero, 1998; Powers, 2000; Karakurt, 2001; Levy and Kelly, 2010) used vignette self-categorization measures of attachment style (Hazan and Shaver, 1987; Bartholomew and Horowitz, 1991). Given the limited variance of categorical measures, we used a more fine-grained dimensional self-report approach as recommended by Ravitz et al. (2010).

Regarding the first aim, we investigate whether (and which) Big Five traits explain variance in romantic jealousy above and beyond attachment dimensions and vice versa. We then depict the interplay among romantic jealousy, the attachment dimensions, as well as the relevant Big Five traits in a path model. Regarding the second aim of our study, this path model is then examined for differences between men and women, individuals in a committed relationship and singles, as well as between individuals with and without prior infidelity experience. Finally, we test whether differences in jealousy between these groups can be explained by Big Five traits and/or attachment dimensions.

## Materials and Methods

### Participants

Participants were recruited *via* flyers, psychology mailing lists, blogs, and social media ads. The online survey was accessed 1,671 times, but data from 653 persons contained mostly missing data and was therefore not considered for further analysis. Data from 130 participants were excluded because they finished the questionnaire in less than 700 s (approximately 11.5 min), which was considered too quick to diligently read and respond to all questions. Another 41 participants were eliminated for not complying with inclusion criteria (under 18 years, never involved in a romantic relationship, polyamory) or because of missing data for age or sex. In 187 cases, missing at random data for single items was replaced with multiple imputations (Rubin, 1987). Visual data screening identified 44 outliers (2.5 standard deviations above or below the sample mean for any variable), with nine outliers within the outcome variable romantic jealousy. There were no significant changes in results after removing outliers, therefore results are presented with these outliers included.

The final sample consisted of 509 women and 338 men ranging in age from 18 to 63 years (*M* = 27.8, SD = 9.6 years). The majority reported to be heterosexual (*N* = 755), 15 participants reported to be homosexual, 61 to be bisexual and another 16 did not provide an answer. The relationship status of 75% was “in a committed relationship or married,” 24% were single, and 1% reported “another” kind of relationship. In a subsample of 630 participants, 37% stated that they made infidelity experiences in the present and/or a previous relationship while 63% stated that they did not make such experiences.

Regarding the highest level of education, 20.9% of the participants had a master’s degree or PhD, 21.3% had a bachelor’s degree, 43.6% had a high school degree, 11.6% finished middle school and an apprenticeship, and 2.7% had a different or no degree. All participants were informed about the study protocol. They provided informed consent prior to their participation by setting a check mark that they have read and understood the study information and agree with the terms of the study. Their anonymity was ensured at all times since their data was never connected with identity-related information. The study was approved by the local ethics committee of the University of Witten/Herdecke (21/2017).

### Measures

#### Big Five Personality Traits

Personality was assessed with the German version of the NEO Five-Factor Inventory (NEO-FFI; Borkenau and Ostendorf, 2008) which measures *neuroticism*, *extraversion*, *openness*, *agreeableness*, and *conscientiousness* with 12 items each. Participants indicate their agreement with each item on a five-point Likert scale from 0 (“strong disagreement”) to 4 (“strong agreement”). Borkenau and Ostendorf (2008) reported internal consistencies for the five scales ranging from *α* = 0.72 to *α* = 0.87.

#### Adult Attachment

Adult attachment was assessed using the German version of the Adult Attachment Scale (AAS; Schmidt et al., 2004) measuring the attachment dimensions *close*, *depend,* and *anxiety*. With five items, the subscale *close* assesses how comfortable a person is with closeness to others. The subscale *depend* measures with six items how much a person trusts in and relies on relevant others. The third subscale *anxiety* consists of five items and refers to fears of being left alone or abandoned. Agreement with each statement was rated on a five-point Likert scale (1 = “totally disagree” to 5 = “totally agree”). All items of the subscale *close* as well as four items of the subscale *depend* were reversed such that higher scores indicated higher comfort with closeness and higher dependency, respectively. The internal consistencies of the subscales range from *α* = 0.72 to *α* = 0.78 (Schmidt et al., 2004).

#### Romantic Jealousy

Jealousy within romantic relationships was measured with the German self-report questionnaire cited by (Schmitt et al., 1995). The questionnaire consists of 15 items, with 10 items focusing on jealousy (perceived intensity and frequency of jealous feelings, cognitive and behavioral effects of jealousy, such as rumination or reproaches) and five items focusing on distrust. Sample jealousy items include “I am bothered when I notice that my partner very much enjoys the company of others,” “I am frequently jealous,” or “I often reproach my partner for being interested in other women/men.” The confirmatory factor analyses by Schmitt et al. (1995) indicated that the distrust items load on a different factor than the jealousy items. To avoid overlap between criterion variance in our jealousy measure and variance in the predictors of personality traits (e.g., agreeableness) or attachment dimensions (e.g., trust in others), we used only the 10 items on jealousy. Participants rated each item on a six-point Likert scale (from 1 = “fits exactly” to 6 = “does not fit at all”) with respect to their current relationship, or their past relationship if they were single.

#### Relationship Variables

Participants stated their relationship status with a multiple-choice item (married with/without children, relationship with/without children, single, other). For the present analyses, relationship status was summarized into three categories; being in a relationship or married, being single, and other.

Due to technical problems at the beginning of data collection, only a subsample of 630 participants responded to questions regarding infidelity experiences. Participants currently living in a relationship were asked to state if their current partner ever cheated on them, with the option to name both sexual and emotional unfaithfulness. All participants were asked about the same infidelity experience in any past relationship. The answers on both items were merged to form two groups of participants with and without former infidelity experiences. Additionally, participants living in a current relationship completed a measure of relationship satisfaction which was not considered in the present analyses.

### Statistical Analyses

All analyses were conducted using RStudio version 1.3.1093. The items of each scale were averaged as test scores before descriptive statistics and correlations between romantic jealousy, the five Big Five traits, and the three attachment dimensions (close, depend, and anxiety) were computed.

Three multiple regressions predicting romantic jealousy were conducted, in which the Big Five (Analysis 1) and the three attachment dimensions (close, depend, and anxiety; Analysis 2) were separately and then jointly (Analysis 3) assessed as independent variables. This analysis resulted in an integrated model of the significant Big Five and attachment predictors of jealousy. The obtained results were used to compute a path analysis on the manifest variables illustrating the direct and indirect effects of the Big Five traits and attachment on jealousy. The maximum likelihood estimator was used. Model/data fit of this path analysis was evaluated by the *χ*^2^ test, the comparative fit index (CFI), the root mean squared error of approximation (RMSEA), and the standardized root mean residual (SRMR). As suggested by Schweizer (2010), the model fit was judged as good (or acceptable) when the *χ*^2^ test was not statistically significant, when the ratio of *χ*^2^ value and its degrees of freedom was less than 2 (or less than 3), when the CFI was larger than 0.950 (or larger than 0.900), when the RMSEA was smaller than 0.050 (or less than 0.080) and when the SRMR was smaller than 0.10.

The path analysis was then repeated but with gender (men vs. women), relationship status (singles vs. individuals in a relationship), and infidelity experiences (with vs. without) as grouping variable, respectively. For each group comparison, the path coefficients were once fixed to be equal in the two respective groups and once they were freely estimated in both groups. The model was assumed to vary between the two groups when the *χ*^2^ difference test indicated significantly better fit of the unrestricted compared to the restricted model and when the CFI of the restricted model was more than 0.01 smaller than the CFI of the unrestricted model (Cheung and Rensvold, 2002). If group differences were found, these were examined more specifically by means of Wald tests on the single path coefficients. Eventually, analyses of covariance (ANCOVAs) were computed to assess whether differences in jealousy between these subgroups could be explained by subgroup differences in personality and attachment dimensions.

As a premise of comparing path coefficients between two groups, metric invariance of the scales between the two groups should be given (Thompson, 2016). We investigated the measurement invariance of the nine scales in the present study (jealousy, Big Five, three attachment dimensions) and found metric invariance for all scales and all group comparisons with the following exceptions. For the openness scale, metric invariance between men and women as well as between singles and individuals in a relationship was only obtained when factor loadings of two items or one item (out of 12) were allowed to vary between the groups, respectively. Thus, only partial metric invariance was given for the openness scale. When comparing individuals with and without infidelity experiences, the extraversion scale from the NEO-FFI and the close scale from the AAS showed only partial metric invariance (factor loadings of two extraversion items and one close item differed between the two groups). In sum, these analyses on measurement invariance showed that metric invariance was largely given.

Since the test scores on the romantic jealousy scale were also analyzed for group differences, we also examined scalar invariance of this scale, which was given for gender differences as well as differences between individuals with and without infidelity experiences. The intercepts of three items, however, differed significantly between singles and individuals in a relationship. Since the scale consists of 10 items, we considered this partial scalar invariance as good enough to interpret potential group differences as differences in the jealousy instead of differences in the responding to single items.

## Results

### Descriptive Statistics, Correlational Analyses, and Group Comparisons

Tables 1 and 2 present the descriptive statistics and Cronbach’s alpha as estimate of the internal consistency for the Big Five personality scales, the three adult attachment scales (comfort with closeness or *close*, dependence on significant others or *depend*, and *anxiety* of being left alone) as well as the romantic jealousy scale in the whole sample and in subsamples by gender, relationship status, and infidelity experience. In the present sample, women, singles, and people with infidelity experience reported higher levels of jealousy than men, individuals in a relationship, and people without infidelity experience, respectively. In addition, men scored higher on the *close* and *depend* dimensions of attachment, and lower on *anxiety*. People in a relationship also scored higher on *close* and lower on *anxiety* than singles, whereas infidelity experience was unrelated to attachment. As for the Big Five, women scored higher than men on neuroticism, whereas men scored higher on extraversion. Singles scored higher on openness and lower on conscientiousness than people in a relationship, whereas infidelity experience was unrelated to the Big Five.

**Table 1:** Mean (M) and standard deviation (SD), Cronbach’s α, and Welch’s *t*-tests evaluating gender differences for the measures of Big Five personality traits, the adult attachment dimensions *close*, *depend,* and *anxiety,* and romantic jealousy.

Measures	Total (*N* = 847)			Men (*N* = 338)		Women (*N* = 509)		*t* (*df*)	Cohen’s *d*
	*M*	SD	*α*	*M*	SD	*M*	SD		
Neuroticism	1.867	0.759	0.88	1.711	0.706	1.971	0.775	−5.054 (766.8)^***^	−0.348
Extraversion	2.301	0.577	0.81	2.364	0.545	2.260	0.595	2.611 (764.48)^**^	0.180
Openness	2.715	0.546	0.75	2.735	0.531	2.702	0.556	0.850 (743.91)	0.059
Agreeableness	2.682	0.515	0.77	2.713	0.470	2.661	0.542	1.481 (787.57)	0.101
Conscientiousness	2.650	0.608	0.85	2.670	0.588	2.636	0.620	0.800 (747.91)	0.056
AAS close	3.523	0.976	0.85	3.639	0.914	3.446	1.009	2.881 (769.11)^**^	0.198
AAS depend	3.813	0.874	0.84	3.903	0.823	3.754	0.902	2.494 (765.36)^*^	0.172
AAS anxiety	2.421	0.874	0.68	2.294	0.855	2.505	0.876	−3.482 (734.14)^***^	−0.243
Jealousy	2.633	1.065	0.90	2.468	1.019	2.743	1.082	−3.758 (750.85)^***^	−0.264

AAS, Adult Attachment Scale.

* *p* < 0.05;

** *p* < 0.01;

*** *p* < 0.001.

**Table 2:** Descriptive statistics for the Big Five personality traits, the adult attachment dimensions *close*, *depend,* and *anxiety,* and romantic jealousy by relationship status and by infidelity experience with *t-*tests assessing group differences.

Measures	In a relationship (*N* = 633)		Single (*N* = 203)		*t* (*df*)	Cohen’s *d*	With infidelity experience (*N* = 232)		Without infidelity experience (*N* = 398)		*t* (*df*)	Cohen’s *d*
	*M*	SD	*M*	SD			*M*	SD	*M*	SD		
Neuroticism	1.867	0.753	1.876	0.786	−0.131 (329.07)	−0.011	1.955	0.774	1.886	0.781	1.078 (486.968)	0.089
Extraversion	2.307	0.581	2.296	0.570	0.224 (346.53)	0.018	2.257	0.610	2.290	0.584	−0.656 (466.054)	−0.054
Openness	2.671	0.544	2.849	0.527	−4.161 (350.65)^***^	−0.330	2.767	0.563	2.703	0.552	1.377 (475.369)	0.114
Agreeableness	2.681	0.511	2.683	0.534	−0.045 (329.09)	−0.004	2.608	0.518	2.682	0.531	−1.715 (493.620)	−0.142
Conscientiousness	2.682	0.600	2.545	0.628	2.729 (328.63)^**^	0.225	2.588	0.615	2.654	0.630	−1.281 (492.695)	−0.106
AAS close	3.573	0.968	3.388	0.998	2.312 (332.64)^*^	0.189	3.416	1.050	3.508	0.958	−1.092 (448.185)	−0.090
AAS depend	3.850	0.864	3.719	0.894	1.830 (331.77)	0.150	3.680	0.921	3.801	0.870	−1.615 (461.180)	−0.133
AAS anxiety	2.390	0.874	2.536	0.869	−2.076 (342.79)^*^	−0.167	2.514	0.944	2.421	0.887	1.218 (459.179)	−0.101
Jealousy	2.589	1.070	2.769	1.040	−2.134 (351.92)^*^	−0.172	2.848	1.176	2.616	1.079	2.466 (450.3)^***^	0.204

AAS, Adult Attachment Scale.

* *p* < 0.05;

** *p* < 0.01;

*** *p* < 0.001.

Table 3 shows the intercorrelations between the Big Five, attachment dimensions, and jealousy. A medium positive correlation was found between jealousy and neuroticism, whereas the correlations between jealousy and the other Big Five traits were small and negative. Jealousy also showed a medium-to-large positive correlation with *anxiety* and small negative correlations with *close* and *depend*. Except for openness, all Big Five traits were related to the three attachment dimensions. Neuroticism correlated negatively with *close* and *depend* but positively with *anxiety*. Extraversion, agreeableness, and conscientiousness correlated positively with *close* and *depend* but negatively with *anxiety*.

**Table 3:** Pearson correlations between jealousy, the Big Five personality traits, the adult attachment dimensions *close*, *depend,* and *anxiety,* romantic jealousy, and age in 847 participants.

	NEO E	NEO O	NEO A	NEO C	AAS C	AAS D	AAS A	Jealousy	Age
NEO N	−0.50^***^	−0.04	−0.23^***^	−0.31^***^	−0.35^***^	−0.50^***^	0.62^***^	0.38^***^	−0.14^***^
NEO E		0.07^*^	0.28^***^	0.24^***^	0.45^***^	0.44^***^	−0.31^***^	−0.19^***^	0.03
NEO O			0.13^***^	−0.13^***^	0.04	0.05	−0.03	−0.18^***^	0.04
NEO A				0.10^**^	0.34^***^	0.46^***^	−0.30^***^	−0.19^***^	0.03
NEO C					0.15^***^	0.21^***^	−0.22^***^	−0.07^*^	0.07^*^
AAS C						0.59^***^	−0.31^***^	−0.20^***^	0.03
AAS D							−0.56^***^	−0.36^***^	0.04
AAS A								0.43^***^	−0.12^***^
Jealousy									−0.15^***^

NEO N, neuroticism; NEO E, extraversion; NEO O, openness; NEO A, agreeableness; NEO C, conscientiousness; AAS C, close; AAD D, depend; AAS A, anxiety.

* *p* < 0.05;

** *p* < 0.01;

*** *p* < 0.001.

### Big Five and Attachment as Predictors of Jealousy

To examine the independent and joint contribution of attachment and the Big Five in explaining individual differences in jealousy, three multiple regression models were run. Since age was negatively related to jealousy, all models were calculated with age as a control variable.

In the first model, the *Big Five* personality traits were entered as predictors of jealousy (see Analysis 1 in Table 4). Altogether, the five personality traits explained 19.4% of the variance in jealousy, but only neuroticism, openness, and agreeableness explained uniquely significant portions of variance, whereas the unique contributions of extraversion and conscientiousness were not statistically significant.

**Table 4:** Linear regression models predicting romantic jealousy in 847 participants.

	*B*	SE (*B*)	*β*	*t*	*p*
*Analysis 1: Big Five traits*					
Age	−0.015	0.003	−0.131	−4.180	<0.001
Neuroticism	0.510	0.052	0.363	9.767	<0.001
Extraversion	0.049	0.068	0.026	0.721	0.471
Openness	−0.286	0.062	−0.146	−4.632	<0.001
Agreeableness	−0.184	0.067	−0.089	−2.735	<0.01
Conscientiousness	0.063	0.058	0.036	1.088	0.277
*R^2^* = 0.194					
*Analysis 2: adult attachment*					
Age	−0.016	0.003	−0.143	−4.691	<0.001
AAS close	0.012	0.041	0.011	0.284	0.776
AAS depend	−0.219	0.053	−0.179	−4.141	<0.001
AAS anxiety	0.389	0.045	0.319	8.638	<0.001
*R^2^* = 0.224					
*Analysis 3: integrated model*					
Age	−0.014	0.003	−0.127	−4.220	<0.001
Neuroticism	0.187	0.055	0.133	3.399	<0.01
Openness	−0.308	0.058	−0.158	−5.272	<0.001
Agreeableness	0.019	0.070	0.009	0.271	0.786
AAS depend	−0.169	0.048	−0.138	−3.480	<0.01
AAS anxiety	0.313	0.050	0.257	6.292	<0.001
*R^2^* = 0.257					

AAS, Adult Attachment Scale.

In the second model (see Analysis 2 in Table 4), the three adult attachment dimensions were examined as predictors accounting for 22.4% of the variance in jealousy. The attachment dimension c*lose* was no unique predictor, but *depend* and *anxiety* explained unique and significant portions of variance in romantic jealousy.

In the third model, the significant predictors of the first two models were entered as predictors (see Analysis 3 in Table 4). The attachment dimensions *anxiety* and *depend* were still significant predictors and their unique contribution to the variance in jealousy only changed marginally compared to the second model. For neuroticism, the beta coefficient was still significant but clearly lowered compared to the first model. The beta coefficient of openness was almost unchanged in the third compared to the first model, while agreeableness lost its predictive power for jealousy.

A comparison of the explained variances of jealousy in the three models showed that attachment and personality traits each explained similar portions of variance (22.4% vs. 19.4%). Furthermore, the third model explained only 3.3% more variance in jealousy as compared to the second model, indicating that most of the explained variance in jealousy was shared between attachment and personality traits. The joint analysis of attachment dimensions and personality traits (Analysis 3) affected the predictive power of the Big Five (particularly neuroticism) more than the predictive power of the attachment dimensions. This finding suggests that the relation between personality traits and jealousy is at least partly mediated by attachment.

In order to better illustrate the interplay between jealousy, attachment, and personality traits, we repeated the third regression analysis by means of a path analysis. Proceeding from the first two regression analyses, we assumed that neuroticism, agreeableness, and openness as well as *depend* and *anxiety* directly predicted jealousy. Furthermore, regressions from neuroticism, agreeableness, and openness on *depend* and *anxiety* were computed to allow for mediation effects of the attachment dimensions on the relationship between personality traits and jealousy. Correlations between the three personality traits as well as between *anxiety* and *depend* were allowed. Path coefficients from agreeableness on jealousy and from openness on *depend* and *anxiety* as well as the correlation between neuroticism and openness did not yield statistical significance. Thus, these coefficients were set to zero.

Age was not included in the path analysis, as the final regression model (Analysis 3 in Table 4) presented earlier yielded nearly the same beta coefficients when age was not entered as a control variable. That is, although higher age predicted lower jealousy, age did not influence the pattern of associations between personality traits, attachment, and jealousy.

**Figure 1:**
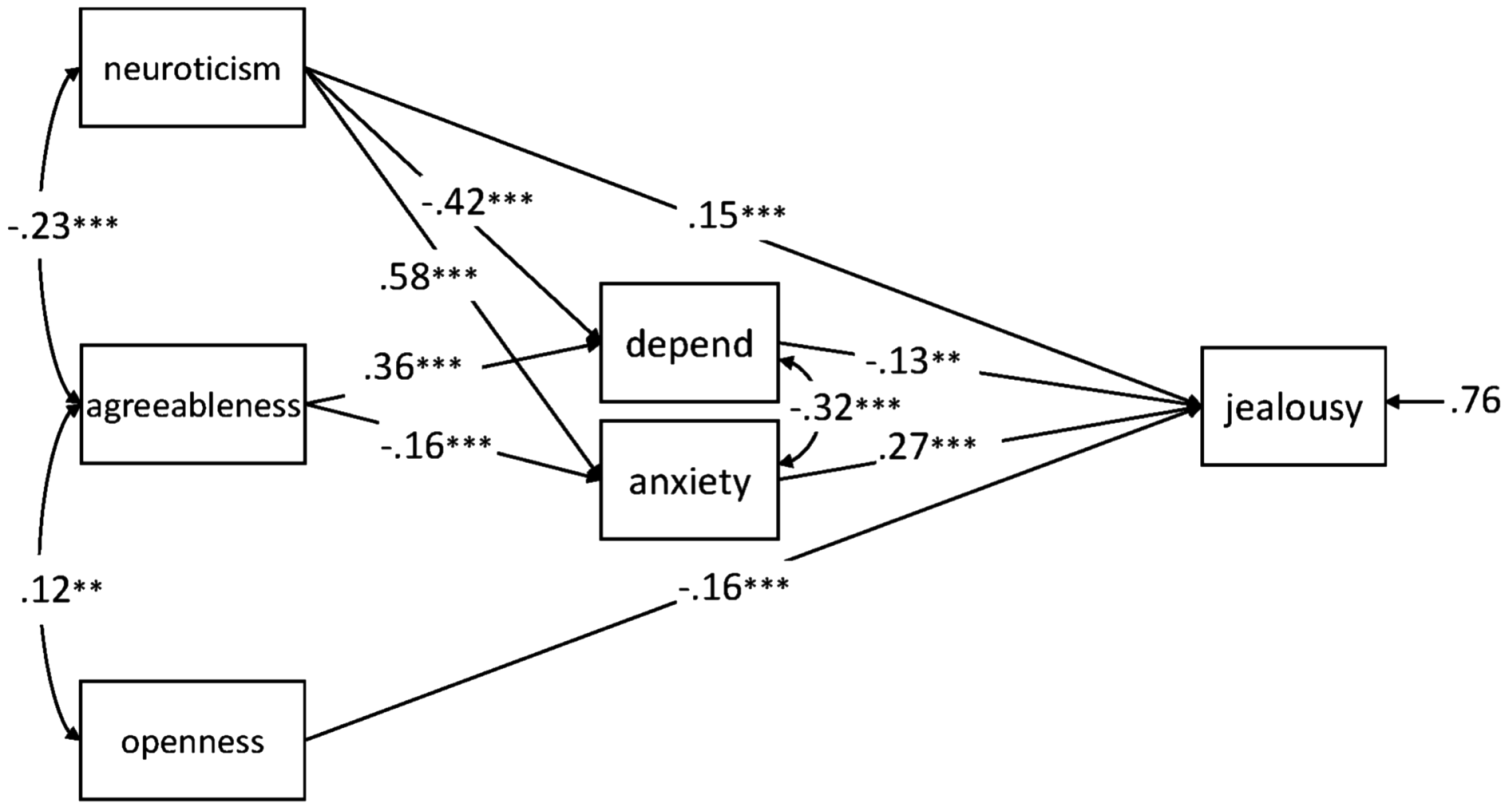
Path analysis with standardized estimates for the relationships between the adult attachment subscales *depend* and *anxiety*, neuroticism, agreeableness, openness, and jealousy in the total sample (*N* = 847). ^**^p < 0.01; ^***^p < 0.001.

The resulting model fit the data well, *χ*^2^(4) = 2.191, *p* = 0.701, CFI = 1.000, RMSEA = 0.000, SRMR = 0.010, AIC = 10,243.740. The significant paths are shown in Figure 1. All indirect effects from neuroticism and agreeableness *via* depend and anxiety on jealousy were statistically significant (all *p*s < 0.01).

### Gender, Relationship Status, and Infidelity Experience as Moderators of the Associations Between Personality, Attachment, and Jealousy

In order to assess whether the association between personality, attachment, and jealousy differed by gender, relationship status, or infidelity experience, the path analysis presented above was recomputed with each of these dichotomous variables as a grouping variable. The zero-order correlations between personality, attachment, and jealousy are reported separately by gender, relationship status, and infidelity experience in Supplementary Tables S1–S3.

#### Gender

**Figure 2:**
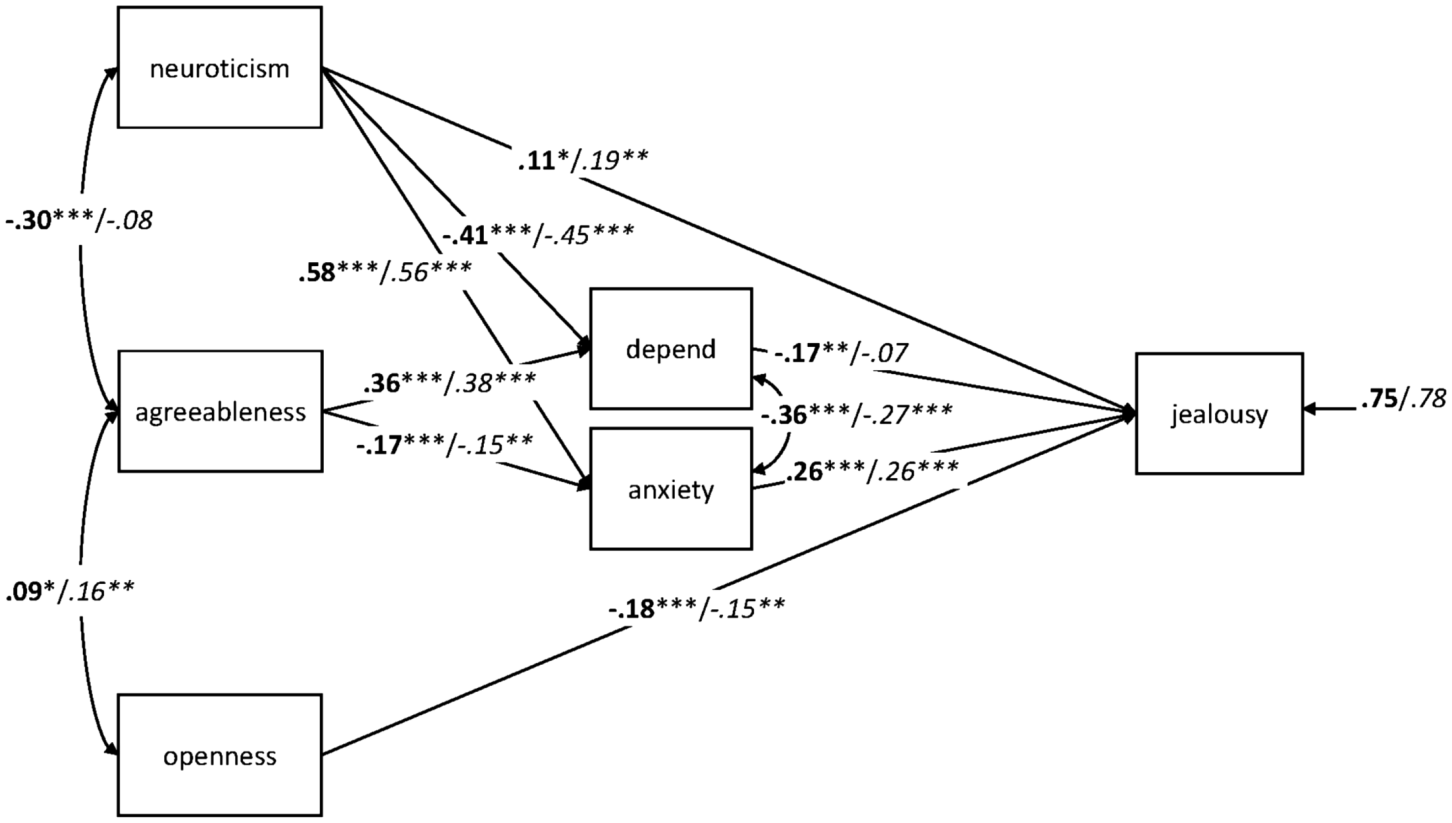
Path analysis with standardized estimates for the relationships between the adult attachment subscales *depend* and *anxiety*, neuroticism, agreeableness, openness, and jealousy in 338 men (italics) and the 509 women (bold). ^*^p < 0.05; ^**^p < 0.01; ^***^p < 0.001.

The path model with gender as grouping variable described the data well, *χ*^2^(8) = 15.376, *p* = 0.052, CFI = 0.994, RMSEA = 0.047, SRMR = 0.031, AIC = 10,242.988. As can be seen in Figure 2, the path coefficients were very similar for men and women. In order to test for significant gender differences, we restricted the path coefficients to be equal in men and women. These constraints resulted in a decrease of the model fit, *χ*^2^(19) = 35.853, *p* = 0.011, CFI = 0.986, RMSEA = 0.046, SRMR = 0.055, AIC = 10,241.465. As indicated by a *χ*^2^ difference test, the model fit was significantly worse compared to the fit of the unconstrained model, Δ*χ*^2^(11) = 20.477, *p* = 0.039. Although the CFI difference with CFI = 0.008 did not exceed the critical value of 0.01, we computed Wald tests for each path to determine sex differences more specifically. The only significant difference between men and women was found in the correlation between neuroticism and agreeableness, *W*(1) = 13.818, *p* < 0.001. When freeing this correlation while keeping all other paths equal in men and women, model fit was good, *χ*^2^(18) = 22.159, *p* = 0.225, CFI = 0.997, RMSEA = 0.023, SRMR = 0.032, AIC = 10,229.772, and not significantly worse than the completely unrestricted model, Δ*χ*^2^(10) = 6.783, *p* = 0.746, ΔCFI = 0.003. It should be noted that the negative effect of *depend* on jealousy was significant in women but not in men. The sex difference of this effect, however, was not significant, *W*(1) = 1.703, *p* = 0.192. Taken together, these results suggest that the path model predicting jealousy from personality and attachment holds for both genders.

Eventually, we examined whether the gender differences in neuroticism, depend, and anxiety (see Table 1) could explain the gender difference in jealousy. For this purpose, we computed an analysis of covariance (ANCOVA) with men and women as two levels of a between-subject factor and neuroticism, depend, and anxiety as covariates. Under consideration of these covariates, the difference in jealousy between men and women just failed to reach statistical significance, *F*(1, 842) = 3.834, *p* = 0.051, *η_p_*^2^ = 0.005, suggesting that the higher scores of women in neuroticism and *anxiety* as well as their lower values on depend at least partly explain their higher scores in jealousy.

#### Relationship Status

**Figure 3:**
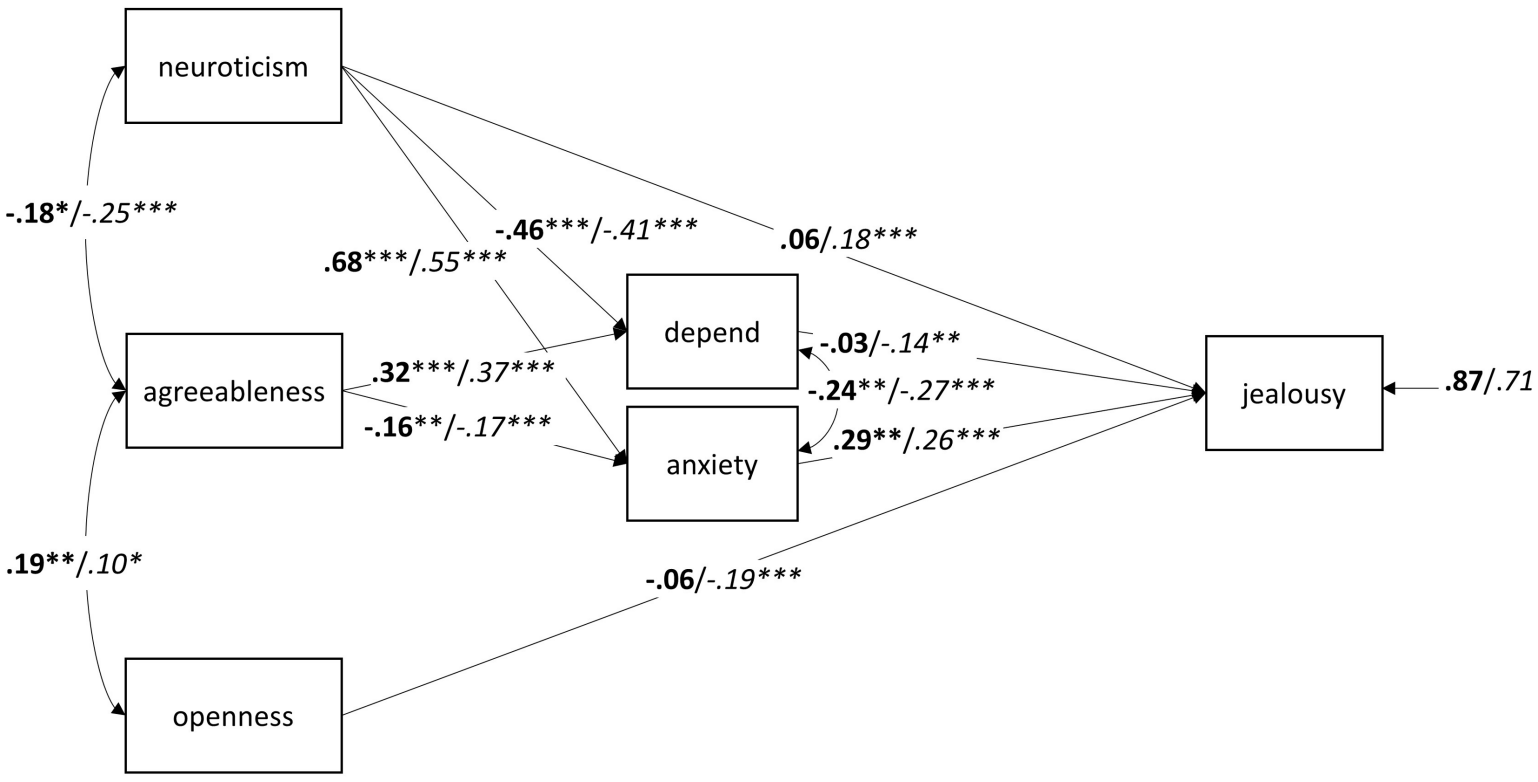
Path analysis with standardized estimates for the relationships between the adult attachment subscales *depend* and *anxiety*, neuroticism, agreeableness, openness, and jealousy in 203 singles (bold) and 633 individuals in a committed relationship (italics). ^*^p < 0.05; ^**^p < 0.01; ^***^p < 0.001.

In order to probe whether the associations between personality, attachment, and jealousy differed by relationship status, the path model depicted in Figure 1 was recomputed with relationship status as grouping variable. The model fit the data well, *χ*^2^(8) = 9.646, *p* = 0.291, CFI = 0.999, RMSEA = 0.022, SRMR = 0.027, AIC = 10,120.177. Constraining the path coefficients to be equal in the two groups (singles vs. individuals in a romantic relationship) led to a decrease in model fit, *χ*^2^(19) = 27.203, *p* = 0.100, CFI = 0.993, RMSEA = 0.032, SRMR = 0.042, AIC = 10,115.735. The difference in the *χ*^2^ fit statistic for the constrained and the unconstrained model did not yield statistical significance, Δ*χ*^2^(11) = 17.557, *p* = 0.092, and the CFI difference of ΔCFI = 0.006 was not larger than 0.01. This result suggested that relationship status did not affect the interplay between personality, attachment, and jealousy. This result was surprising given that the paths from neuroticism, openness, and *depend* on jealousy were statistically significant in individuals in a romantic relationship but not in singles. Wald tests, however, supported the overall results and indicated that neither the path from neuroticism to jealousy, *W*(1) = 1.545, *p* = 0.214, the path from openness to jealousy, *W*(1) = 3.356, *p* = 0.067, nor the path from depend on jealousy, *W*(1) = 1.704, *p* = 0.192, differed significantly between the two groups (Figure 3).

To investigate whether individuals in a romantic relationship and singles differed in jealousy due to their differences in openness and anxiety (see Table 2), we computed a one-way ANCOVA with romantic jealousy as dependent variable, relationship status as a group factor, and openness and anxiety as two covariates. The result of this ANCOVA indicated that the difference between singles and individuals in a current relationship was still significant when controlling for the covariates, *F*(1, 832) = 4.678, *p* = 0.031, *η_p_*^2^ = 0.006, suggesting that differences in openness and anxiety could not explain the higher jealousy of singles.

#### Infidelity Experience

**Figure 4:**
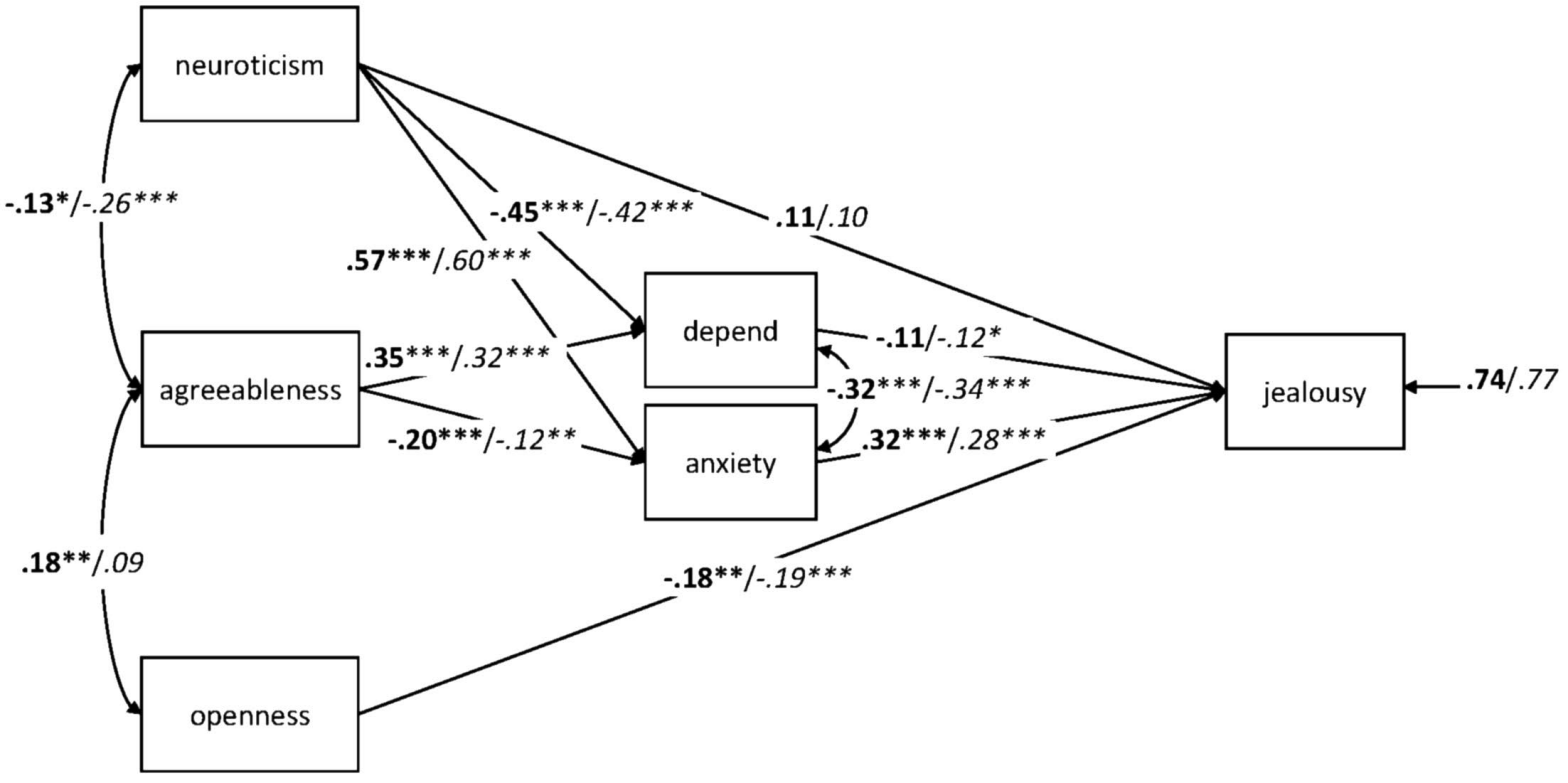
Path analysis with standardized estimates for the relationships between the adult attachment subscales *depend* and *anxiety*, neuroticism, agreeableness, openness, and jealousy in 232 individuals with previous infidelity experiences (bold) and 398 individuals without such experiences (italics). ^*^p < 0.05; ^**^p < 0.01; ^***^p < 0.001.

Using the same analytic strategy as for gender and relationship status, Figure 4 provides the results of the path analysis with separate coefficients for 232 individuals with and 398 individuals without previous experiences of infidelity by their (former) partner. The model fit the data well, *χ*^2^(8) = 16.277, *p* = 0.039, CFI = 0.991, RMSEA = 0.057, SRMR = 0.032, AIC = 7,906.201.

When all paths were set equal for the two groups, the model fit was still good, *χ*^2^(19) = 23.345, *p* = 0.222, CFI = 0.995, RMSEA = 0.027, SRMR = 0.041, AIC = 7,891.269. This fit was not significantly worse compared to the fit of the unconstrained model as indicated by a non-significant *χ*^2^ difference test, Δ*χ*^2^(11) = 7.068, *p* = 0.794, and a CFI difference of ΔCFI = 0.004. This result was plausible given the high similarity of the path coefficients in Figure 4 for individuals with and without former infidelity experience. Given that none of the present predictors of romantic jealousy differed significantly between the two groups, the computation of an ANCOVA with personality traits and attachment dimensions as covariates was not appropriate. Thus, personality and/or attachment dimensions cannot explain why individuals with infidelity experience scored higher on jealousy than individuals without infidelity experience.

## Discussion

Jealousy is an important characteristic of romantic relationships. Although romantic jealousy has been widely researched, relatively little is known about how stable personality dispositions predict the habitual level of jealousy that people experience when being in a relationship. The main aim of the present study was therefore to jointly assess two sets of personality dispositions—the Big Five traits and adult attachment dimensions—as predictors of romantic jealousy in the current or in a past relationship, resulting in a path model illustrating the shared and unique contributions of each trait or dimension. The second aim was to examine whether the paths between the Big Five, attachment, and jealousy differed by gender, relationship status, or infidelity experience in order to gain a deeper understanding of the stability and generalizability of the personality—jealousy link.

With respect to the first aim, regression analyses showed that the Big Five and the three adult attachment dimensions (*close*, *depend*, and *anxiety*) predicted individual differences in jealousy similarly well, with attachment explaining slightly more variance. In addition, the variance explained increased only slightly when the significant traits or dimensions from each of the two variable sets were jointly assessed as predictors of jealousy. This suggests that much of the explained variance is shared between the Big Five and attachment. These results are consistent with previous studies examining the correlations between the Big Five (or single Big Five traits) and jealousy (e.g., Melamed, 1991; Buunk, 1997; Wade and Walsh, 2008), between the Big Five and attachment (e.g., Noftle and Shaver, 2006), and between attachment and jealousy (e.g., Sharpsteen and Kirkpatrick, 1997), which all pointed out conceptual similarities between these constructs and generally found small to medium associations among them.

As a novel contribution to the field, the path model shown in Figure 1 displays the direct and indirect contributions of the Big Five and attachment to jealousy. Higher levels of neuroticism positively predicted jealousy, both directly as well as indirectly *via* the two attachment dimensions depend (i.e., *via* lower levels of trust toward and reliance on others) and anxiety (i.e., *via* higher levels of anxiety of being abandoned and rejected). Lack of trust and high levels of fear correspond well to some of the core facets of neuroticism, such as anxiety, self-consciousness, and vulnerability (Costa and McCrae, 2012). In the attachment—jealousy model by Harris and Darby (2010), less trust and higher fear of being abandoned are related to the first stage of jealousy, that is, having a lower threshold for perceiving someone else as a rival. The unique contribution of neuroticism above and beyond these facets might come from other facets, such as anger and impulsivity (Costa and McCrae, 2012), which are linked to the second stage of jealousy (the reaction to an actual rival). People with high levels of dispositional anger and impulsivity might show stronger jealous reactions toward the partner and/or the rival (Harris and Darby, 2010).

The second Big Five trait to predict higher jealousy was low agreeableness, although this effect was smaller and fully mediated by the attachment dimensions *depend* and *anxiety*. Again, this association is plausible given that low agreeableness is characterized by a lack of trust and a general tendency to believe that others are malevolent. The third significant personality predictor in the path model was low openness to experience, which explained higher jealousy independently of attachment with a small effect size. Although past studies did not find this association (Wade and Walsh, 2008), it might be explained through the facets *openness to feelings*, which has been associated with more successful emotion regulation and thinking more positively, as well as *openness to ideas* which allows for the more flexible generation of explanations (Nekljudova, 2019). People high in openness might reason more flexibly about their partner’s potentially “suspicious” behaviors and might be better able to regulate their response to a relationship threat. In addition, people high in openness more often receive mate poaching attempts and therefore might be more confident that the presence of a rival does not necessarily mean that their relationship is at risk (Schmitt and Buss, 2001).

Generally, these findings confirm past findings that neuroticism is the most important Big Five trait when explaining both attachment and jealousy (e.g., Buunk, 1997). But the present study adds to the past literature that openness also explains unique variance in jealousy that is not covered by adult attachment and that agreeableness contributes to jealousy indirectly *via* more secure and less avoidant attachment styles. This supports the importance of broad personality dispositions in the specific life domain of romantic relationships (Costa and McCrae, 2012). Interestingly, the Big Five trait extraversion, which affects interpersonal relationships more generally (e.g., Malouff et al., 2010), was also related to romantic jealousy in the correlational analyses. However, when all Big Five traits were jointly considered, this effect disappeared indicating that the extraversion—romantic jealousy link is probably indirect.

Among the adult attachment dimensions, *anxiety* (i.e., feelings of inadequacy and fear of being abandoned) was the strongest predictor of high jealousy, whereas a low tendency to rely on and trust others (*depend*) had a smaller effect. Somewhat surprisingly, the dimension *close* (feeling comfortable with close relationships) did not independently predict jealousy, which might be explained by the high intercorrelation with *depend*. Attachment avoidance has been proposed as the common factor underlying low values in both *depend* and *close* (Collins and Feeney, 2000). As such, the present results correspond to earlier findings using self-categorization measures of attachment, in which associations with jealousy were less clear-cut for avoidantly as compared to securely or anxiously attached individuals (Harris and Darby, 2010).

Given the high conceptual overlap primarily between attachment and jealousy, it was surprising that attachment dimensions and Big Five personality traits explained only about 25% of the variance in romantic jealousy. Thus, 75% of the individual differences in romantic jealousy are probably caused by other factors. In would be interesting for future studies to investigate the influence of relationship-related variables, such as relationship satisfaction, duration, and quality as well as further situational and environmental factors, such as socioeconomic status, dependency on the partner, or the presence and number of children.

With respect to the second aim, our results showed that the path model illustrating the links between the Big Five, attachment, and jealousy remained virtually unchanged when gender, relationship status, or partners’ (known) infidelity were assessed as moderators. That is, personality traits and attachment dimensions predicted jealousy equally for women and men, people in a relationship and singles, as well as people with and without infidelity experiences. This is particularly interesting because the mean levels of jealousy, and partly the mean levels on some personality variables, differed between these groups.

In line with previous studies using self-report measures of jealousy (e.g., Valentova et al., 2020), women expressed higher jealousy than men in the present sample, and this difference was at least partly explained by gender differences in broad personality and attachment dimensions (higher neuroticism and *anxiety* and lower *depend* values in women). However, this result might not generalize to more specific aspects of jealousy, such as sexual vs. emotional jealousy (Edlund and Sagarin, 2017) or to different types of jealousy measures (e.g., scenario measures). Whereas women tend to report higher emotional jealousy, men tend to express more sexual jealousy, and these associations are further qualified by sexual orientation (Valentova, 2019).

Somewhat surprisingly, in the present study singles reported higher levels of jealousy with respect to their last relationship as compared to people currently living in a relationship. Given that retrospective measures often yield less intense reports of jealousy than current measures (Edlund and Sagarin, 2017), we expected the opposite result (see also Valentova et al., 2020). In our sample, singles scored higher on openness, which was associated with less jealousy, but they also reported more attachment anxiety, which predicted more jealousy. This pattern did not explain singles’ higher level of jealousy, but it raises different possible explanations that could be explored in future longitudinal studies. For instance, some of the singles in our sample may have recently been abandoned by their partners and have developed higher levels of anxiety, whereas others may be single because of their higher fear of being rejected or because of their higher level of openness to new experiences.

We also found people whose current or past partner had been (sexually) unfaithful to report higher levels of jealousy as compared to people without such infidelity experiences. Previous research has found this relationship mostly in men, whereas women tend to be more jealous in response to emotional infidelity (e.g., Edlund et al., 2006). Importantly, these two participant groups did not differ in the Big Five or attachment, suggesting that there is no personality pattern that increases the likelihood to be cheated on. Vice versa, this finding implies that experiences like infidelity affect more proximal feelings and behaviors in romantic relationships (i.e., jealousy), but not more stable dispositions like personality or attachment.

In sum, gender, relationship status, and infidelity experiences were found to be associated with differences in romantic jealousy. Despite these mean differences, however, the pattern of associations of romantic jealousy with personality traits and attachment dimensions seems to be unaffected by gender, relationship status, and infidelity experiences. This finding points to a stable network of personal characteristics underlying romantic jealousy. While corresponding mean differences in personality traits and attachment dimensions can even explain gender differences in romantic jealousy, they cannot explain differences between individuals in a romantic relationship and singles nor between individuals with and without infidelity experiences. These latter differences, therefore, are likely caused by more situational factors as additional and apparently independent sources of individual differences in romantic jealousy besides personality traits and attachment dimensions.

The present study has some strengths as well as various limitations. A strength is that to our knowledge, this is the first study to jointly and comprehensively assess basic personality and attachment dimensions as predictors of romantic jealousy, resulting in a path model which contributed to our understanding of the shared and unique effects of the different dimensions. In addition, the study was based on a large and varied sample, adding to the generalizability of the established predictive model of romantic jealousy with regard to gender, relationship status, and infidelity experience. Other variables, however, such as socioeconomic status, level of education, and sexual orientation, were not considered because of lacking information or too small subsamples. A further limitation is the rather global assessment of jealousy which did not allow for a separate analysis of emotional, cognitive, behavioral, sexual, or other types of jealousy. Further, all variables were assessed *via* self-report which is prone to problems like social desirability or self-deception. Future studies should consider incorporating partner ratings of jealousy as well as other types of measures (e.g., scenario tasks) to further validate the associations between personality, attachment, and jealousy found here. In addition, the present study was cross-sectional and hence, our path model does not reflect the causal directions. As described above, the path model seems to make sense conceptually, with the most basic and broad personality dimensions affecting more relationship-specific attachment dimensions, which in turn predict a specific and potentially less stable relationship variable (i.e., jealousy). Longitudinal studies, however, would be needed to interpret the network, demonstrated in the present study, in a causal manner.

## Data Availability Statement

The raw data supporting the conclusions of this article will be made available by the authors, without undue reservation.

## Ethics Statement

The studies involving human participants were reviewed and approved by Ethikkommission der Universität Witten/Herdecke. The patients/participants provided their written informed consent to participate in this study.

## Author Contributions

All authors contributed to conception and design of the study. MR and PT organized the database. MR and ST performed the statistical analyses. MR wrote the first draft of the manuscript. KS and ST wrote sections of the manuscript. All authors contributed to manuscript revision, read, and approved the submitted version.

## Funding

Open access funding provided by University of Bern.

## Conflict of Interest

The authors declare that the research was conducted in the absence of any commercial or financial relationships that could be construed as a potential conflict of interest.

## Publisher’s Note

All claims expressed in this article are solely those of the authors and do not necessarily represent those of their affiliated organizations, or those of the publisher, the editors and the reviewers. Any product that may be evaluated in this article, or claim that may be made by its manufacturer, is not guaranteed or endorsed by the publisher.

## References

[ref1] Altmann T. Roth M. (2020). Individual differences in the preference for cross-sex friendship (heterosociality) in relation to personality. Personal. Individ. Differ. 157:109838. doi: 10.1016/j.paid.2020.109838

[ref2] Bartholomew K. Horowitz L. M. (1991). Attachment styles among young adults: a test of a four-category model. J. Pers. Soc. Psychol. 61, 226–244. doi: 10.1037/0022-3514.61.2.226, PMID: 1920064

[ref3] Borkenau P. Ostendorf F. (2008). NEO-FFI – NEO-Fünf-Faktoren-Inventar nach Costa und McCrae. Göttingen: Hogrefe.

[ref4] Bowlby J. (1969, 1982). Attachment and Loss: Attachment. New York: Basic Books.

[ref5] Buunk B. P. (1982). Anticipated sexual jealousy: its relationship to self-esteem, dependency, and reciprocity. Personal. Soc. Psychol. Bull. 8, 310–316. doi: 10.1177/0146167282082019

[ref6] Buunk B. P. (1995). Sex, self-esteem, dependency and extradyadic sexual experience as related to jealousy responses. J. Soc. Pers. Relat. 12, 147–153. doi: 10.1177/0265407595121011

[ref7] Buunk B. P. (1997). Personality, birth order and attachment styles as related to various types of jealousy. Personal. Individ. Differ. 23, 997–1006. doi: 10.1016/S0191-8869(97)00136-0

[ref8] Cheung G. W. Rensvold R. B. (2002). Evaluating goodness-of-fit indexes for testing measurement invariance. Struct. Equ. Model. 9, 233–255. doi: 10.1207/S15328007SEM0902_5

[ref9] Collins N. L. Feeney B. C. (2000). A safe haven: an attachment theory perspective on support seeking and caregiving in intimate relationships. J. Pers. Soc. Psychol. 78, 1053–1073. doi: 10.1037/0022-3514.78.6.1053, PMID: 10870908

[ref10] Collins N. L. Read S. J. (1990). Adult attachment, working models, and relationship quality in dating couples. J. Pers. Soc. Psychol. 58, 644–663. doi: 10.1037/0022-3514.58.4.644, PMID: 14570079

[ref11] Costa P. T. McCrae R. R. (1992). Revised NEO Personality Inventory (NEO-PI-R) and NEO Five-Factor Inventory (NEO-FFI) Professional Manual. Odessa, FL: Psychological Assessment Resources.

[ref12] Costa P. T. McCrae R. R. (2012). “The five-factor model, five-factor theory, and interpersonal psychology,” in Handbook of Interpersonal Psychology. eds. Horowitz L. M. Strack S. (Hoboken, New Jersey: Wiley & Sons, Inc.), 91–104.

[ref13] Dijkstra P. Barelds D. P. H. (2008). Self and partner personality and responses to relationship threats. J. Res. Pers. 42, 1500–1511. doi: 10.1016/j.jrp.2008.06.008

[ref14] Dugosh J. W. (2000). On predicting relationship satisfaction from jealousy: the moderating effects of love. Curr. Res. Soc. Psychol. 5, 254–263.

[ref15] Edlund J. E. Heider J. D. Scherer C. R. Farc M.-M. Sagarin B. J. (2006). Sex differences in jealousy in response to actual infidelity. Evol. Psychol. 4:147470490600400. doi: 10.1177/147470490600400137

[ref16] Edlund J. E. Sagarin B. J. (2017). Sex differences in jealousy: a 25-year retrospective. Adv. Exp. Soc. Psychol. 55, 259–302. doi: 10.1016/bs.aesp.2016.10.004

[ref17] Ein-Dor T. Perry-Paldi A. Hirschberger G. Birnbaum G. E. Deutsch D. (2015). Coping with mate poaching: gender differences in detection of infidelity-related threats. Evol. Hum. Behav. 36, 17–24. doi: 10.1016/j.evolhumbehav.2014.08.002

[ref18] Gehl B. K. (2010). Personality antecedents of the experience and expression of romantic jealousy. Unpublished PhD thesis. University of Iowa.

[ref19] Guerrero L. K. (1998). Attachment-style differences in the experience and expression of romantic jealousy. Pers. Relat. 5, 273–291. doi: 10.1111/j.1475-6811.1998.tb00172.x

[ref20] Harris C. R. (2003). A review of sex differences in sexual jealousy, including self-report data, psychophysiological responses, interpersonal violence and morbid jealousy. Personal. Soc. Psychol. Rev. 7, 102–128. doi: 10.1207/S15327957PSPR0702_102-128, PMID: 12676643

[ref21] Harris C. R. Darby R. S. (2010). “Jealousy in adulthood,” in Handbook of Jealousy: Theory, Research, and Multidisciplinary Approaches. eds. Hart L. Legerstee M. (Chichester: Wiley Blackwell), 547–571.

[ref22] Hart S. Legerstee M. (2010). Handbook of Jealousy. Theory, Research, and Multidisciplinary Approaches. Chichester: Wiley-Blackwell.

[ref23] Hazan C. Shaver P. (1987). Romantic love conceptualized as an attachment process. J. Pers. Soc. Psychol. 52, 511–524. doi: 10.1037/0022-3514.52.3.511, PMID: 3572722

[ref24] Karakurt G. (2001). The impact of adult attachment styles on romantic jealousy. Unpublished Master’s Thesis. Ankara, Turkey: Middle East Technical University.

[ref25] Karakurt G. (2012). The interplay between self-esteem, feeling of inadequacy, dependency, and romantic jealousy as a function of attachment processes among Turkish college students. Contemp. Fam. Ther. 34, 334–345. doi: 10.1007/s10591-012-9185-7

[ref26] Koenig J. L. Barry R. A. Kochanska G. (2010). Rearing difficult children: parents’ personality and children’s proneness to anger as predictors of future parenting. Parent. Sci. Pract. 10, 258–273. doi: 10.1080/15295192.2010.492038, PMID: 21243035 PMC3018753

[ref27] Levy K. N. Kelly K. M. (2010). Sex differences in jealousy: a contribution from attachment theory. Psychol. Sci. 21, 168–173. doi: 10.1177/0956797609357708 20424039

[ref28] Malouff J. M. Thorsteinsson E. B. Schutte N. S. Bhullar N. Rooke S. E. (2010). The five-factor model of personality and relationship satisfaction of intimate partners: a meta-analysis. J. Res. Pers. 44, 124–127. doi: 10.1016/j.jrp.2009.09.004

[ref29] Marshall T. C. Bejanyan K. Di Castro G. Lee R. A. (2013). Attachment styles as predictors of Facebook-related jealousy and surveillance in romantic relationships. Pers. Relat. 20, 1–22. doi: 10.1111/j.1475-6811.2011.01393.x

[ref30] McCrae R. R. Costa P. T. (2008). “The five-factor theory of personality,” in Handbook of Personality – Theory and Research. 3rd Edn. eds. John O. P. Robins R. W. Pervin L. A. (New York: Guilford Press).

[ref31] Melamed T. (1991). Individual differences in romantic jealousy: the moderating effect of relationship characteristics. Eur. J. Soc. Psychol. 21, 455–461. doi: 10.1002/ejsp.2420210508

[ref32] Mikulincer M. Shaver P. R. (2007). Attachment in Adulthood: Structure, Dynamics, and Change. New York: Guilford Press.

[ref33] Nekljudova S. V. (2019). Six aspects of openness to experience. J. Psychol. Clin. Psychiatry 10, 78–81. doi: 10.15406/jpcpy.2019.10.00632

[ref34] Noftle E. E. Shaver P. R. (2006). Attachment dimensions and the big five personality traits: associations and comparative ability to predict relationship quality. J. Res. Pers. 40, 179–208. doi: 10.1016/j.jrp.2004.11.003

[ref35] Powers A. M. (2000). The effects of attachment style and jealousy on aggressive behavior against a partner and a rival. Diss. Abstr. Int. 61:3325.

[ref36] Ravitz P. Maunder R. Hunter J. Sthankiya B. Lancee W. (2010). Adult attachment measures: a 25-year review. J. Psychosom. Res. 69, 419–432. doi: 10.1016/j.jpsychores.2009.08.006, PMID: 20846544

[ref37] Rubin D. B. (1987). Multiple Imputation for Nonresponse in Surveys. New York: Wiley & Sons.

[ref001] Schweizer K. (2010). Some guidelines concerning the modeling of traits and abilities in test construction. Eur. J. Psychol. Assess. 26, 1–2. doi: 10.1027/1015-5759/a000001

[ref38] Schmidt S. Strauß B. Höger D. Brähler E. (2004). Die Adult Attachment Scale (AAS) – Teststatistische Prüfung und Normierung der deutschen Version. Psychother. Psychosom. Med. Psychol. 54, 375–382. doi: 10.1055/s-2003-815000 15343479

[ref39] Schmitt D. P. Buss D. M. (2001). Human mate poaching: tactics and temptations for infiltrating existing mateships. J. Pers. Soc. Psychol. 80, 894–917. doi: 10.1037/0022-3514.80.6.894, PMID: 11414373

[ref40] Schmitt M. J. Falkenau K. Montada L. (1995). Zur Messung von Eifersucht über stellvertretende Emotionsbegriffe und zur Bereichsspezifität der Eifersuchtsneigung. Diagnostica 41, 131–149.

[ref41] Sharpsteen D. J. Kirkpatrick L. A. (1997). Romantic jealousy and adult romantic attachment. J. Pers. Soc. Psychol. 72, 627–640. doi: 10.1037/0022-3514.72.3.627, PMID: 9120787

[ref42] Shaver P. R. Brennan K. A. (1992). Attachment styles and the “big five” personality traits: their connections with each other and with romantic relationship outcomes. Personal. Soc. Psychol. Bull. 18, 536–545. doi: 10.1177/0146167292185003

[ref43] Sheets V. L. Fredendall L. L. Claypool H. M. (1997). Jealousy evocation, partner reassurance, and relationship stability: an exploration of the potential benefits of jealousy. Evol. Hum. Behav. 18, 387–402. doi: 10.1016/S1090-5138(97)00088-3

[ref44] Tagler M. J. (2010). Sex differences in jealousy: comparing the influence of previous infidelity among college students and adults. Soc. Psychol. Personal. Sci. 1, 353–360. doi: 10.1177/1948550610374367

[ref45] Thompson M. S. (2016). “Assessing measurement invariance of scales using multiple-group structural equation modeling,” in Principles and Methods of Test Construction. eds. Schweizer K. DiStefano C. (Göttingen: Hogrefe).

[ref46] Valentova J. V. (2019). “Sex differences in jealousy,” in Encyclopedia of Evolutionary Psychological Science. eds. Shackelford T. K. Weekes-Shackelford V. A. (Cham: Springer), 1–4.

[ref47] Valentova J. V. de Moraes A. C. Varella M. A. C. (2020). Gender, sexual orientation and type of relationship influence individual differences in jealousy: a large Brazilian sample. Personal. Individ. Differ. 157:109805. doi: 10.1016/j.paid.2019.109805

[ref48] Varga C. M. Gee C. B. Munro G. (2011). The effects of sample characteristics and experience with infidelity on romantic jealousy. Sex Roles 65, 854–866. doi: 10.1007/s11199-011-0048-8

[ref49] Wade T. J. Walsh H. (2008). Does the Big-5 relate to jealousy, or infidelity reactions? J. Soc. Evol. Cult. Psychol. 2, 133–143. doi: 10.1037/h0099349

[ref50] Wegner R. Roy A. R. K. Gorman K. R. Ferguson K. (2018). Attachment, relationship communication style and the use of jealousy induction techniques in romantic relationships. Personal. Individ. Differ. 129, 6–11. doi: 10.1016/j.paid.2018.02.033

[ref51] White G. L. (1981). Jealousy and partner's perceived motives for attraction to a rival. Soc. Psychol. Q. 44, 24–30. doi: 10.2307/3033859

