# Supplementary material for: Adult Attachment and Personality as Predictors of Jealousy in Romantic Relationships
Source: Front Psychol. 2022 Apr 12;13:861481. doi: 10.3389/fpsyg.2022.861481 (PMC9039013; doi:10.3389/fpsyg.2022.861481)
Supplement: Supplementary file 1 [file Data_Sheet_1.pdf]

Supplementary Material for:

Adult attachment and personality as predictors of jealousy in romantic relationships

Table S1.

Pearson correlations between the Big Five personality traits, the adult attachment dimensions *close*, *depend* and *anxiety* and romantic jealousy in 338 men (above the diagonal) and 509 women (below the diagonal).

	**NEO N**	**NEO E**	**NEO O**	**NEO A**	**NEO C**	**AAS C**	**AAS D**	**AAS A**	**Jealousy**
NEO N		-.41***	.07	-.07	-.24***	-.34***	-.47***	.58***	.37***
NEO E	-.54***		.11	.17***	.20***	.35***	.38***	-.24***	-.15**
NEO O	-.10	.05		.15***	-0.12*	.02	.03	.11*	-.08
NEO A	-.31***	.33***	.11*		.05	.30***	.41***	-.19***	-.11*
NEO C	-.36***	.26***	-.14***	.12***		.14*	.17***	-.17***	-.03
AAS C	-.35***	.49***	.05	.36***	.15***		.57***	-.27***	-.15**
AAS D	-.51***	.47***	.05	.48***	.23***	.60***		-.50***	-.30***
AAS A	.63***	-.34***	-.11*	-.35***	-.24***	-.33***	-.60***		.40***
Jealousy	.37***	-.20***	-.22***	-.22***	-.09*	-.21***	-.39***	.44***	

Note. NEO N = neuroticism; NEO E = extraversion; NEO O = openness; NEO A = agreeableness; NEO C = conscientiousness; AAS C = *close*; AAD D = depend; AAS A = *anxiety*; Jealousy = romantic jealousy. * $p < .05$. ** $p < .01$. *** $p < .001$.

Table S2.

Pearson correlations between the Big Five personality traits, the adult attachment dimensions *close*, *depend* and *anxiety* and romantic jealousy in 203 singles (above the diagonal) and 633 individuals in a romantic relationship (below the diagonal).

	**NEO N**	**NEO E**	**NEO O**	**NEO A**	**NEO C**	**AAS C**	**AAS D**	**AAS A**	**Jealousy**
NEO N		-.54***	.12	-.16*	-.36***	-.35***	-.51***	.70***	.28***
NEO E	-.49***		-.03	.20***	.27***	.39***	.48***	-.40***	-.15*
NEO O	-.10*	.10**		.17*	-.17*	-.03	-.02	.06	-.04
NEO A	-.26***	.31***	.12***		.16*	.22***	.39***	-.26***	-.13
NEO C	-.30***	.23***	-.10**	.08*		.16*	.22***	-.23***	.01
AAS C	-.36***	.47***	.09*	.39***	.14***		.58***	-.26***	-.09
AAS D	-.51***	.44***	.09*	.48***	.21***	.60***		-.54***	-.22**
AAS A	.59***	-.28***	-.06	-.31***	-.20***	-.33***	-.57***		.35***
Jealousy	.42***	-.20***	-.24***	-.20***	-.09*	-.23***	-.40***	.46***	

Note. NEO N = neuroticism; NEO E = extraversion; NEO O = openness; NEO A = agreeableness; NEO C = conscientiousness; AAS C = *close*; AAD D = depend; AAS A = *anxiety*; Jealousy = romantic jealousy.
* $p < .05$. ** $p < .01$. *** $p < .001$.

Table S3.

Pearson correlations between the Big Five personality traits, the adult attachment dimensions *close*, *depend* and *anxiety* and romantic jealousy in 398 individuals without (above the diagonal) and 232 individuals with infidelity experience (below the diagonal).

	**NEO N**	**NEO E**	**NEO O**	**NEO A**	**NEO C**	**AAS C**	**AAS D**	**AAS A**	**Jealousy**
NEO N		-.56***	-.01	-.26***	-.31***	-.36***	-.50***	.63***	.34***
NEO E	-.43***		.04	.31***	.26***	.41***	.40***	-.32***	-.19***
NEO O	-.18**	.18**		.09	-.20***	.00	.00	.08	-.17***
NEO A	-.16*	.26***	.20**		.05	.31***	.43***	-.27***	-.19***
NEO C	-.33***	.15*	-.08	.04		.15**	.19***	-.22***	-.04
AAS C	-.30***	.50***	.15*	.38***	.09		.59***	-.33***	-.23***
AAS D	-.50***	.48***	.18**	.42***	.22**	.58***		-.56***	-.33***
AAS A	.60***	-.28***	-.15*	-.30***	-.20**	-.22***	-.57***		.40***
Jealousy	.38***	-.14*	-.27***	-.14*	-.07	-.09	-.38***	.47***	

Note. NEO N = neuroticism; NEO E = extraversion; NEO O = openness; NEO A = agreeableness; NEO C = conscientiousness; AAS C = *close*; AAD D = depend; AAS A = *anxiety*; Jealousy = romantic jealousy.
* $p < .05$. ** $p < .01$. *** $p < .001$.
